# Effects of Dietary Components on Testosterone Metabolism via UDP-Glucuronosyltransferase

**DOI:** 10.3389/fendo.2013.00080

**Published:** 2013-07-08

**Authors:** Carl Jenkinson, Andrea Petroczi, Declan P. Naughton

**Affiliations:** ^1^School of Life Sciences, Kingston University, Kingston upon Thames, UK

**Keywords:** UGT2B17, inhibition, testosterone, glucuronidation, green tea, red wine, catechins, flavonoids

## Abstract

The potential interference in testosterone metabolism through ingested substances has ramifications for: (i) a range of pathologies such as prostate cancer, (ii) medication contra-indications, (iii) disruption to the endocrine system, and (iv) potential confounding effects on doping tests. Conjugation of anabolic steroids during phase II metabolism, mainly driven by UDP-glucuronosyltransferase (UGT) 2B7, 2B15, and 2B17, has been shown to be impaired *in vitro* by a range of compounds including xenobiotics and pharmaceuticals. Following early reports on the effects of a range of xenobiotics on UGT activity *in vitro*, the work was extended to reveal similar effects with common non-steroidal anti-inflammatory drugs. Notably, recent studies have evidenced inhibitory effects of the common foodstuffs green tea and red wine, along with their constituent flavonoids and catechins. This review amalgamates the existing evidence for the inhibitory effects of various pharmaceutical and dietary substances on the rate of UGT glucuronidation of testosterone; and evaluates the potential consequences for health linked to steroid levels, interaction with treatment drugs metabolized by the UGT enzyme and steroid abuse in sport.

## Introduction

As a major route for excretion of exogenous and endogenous compounds, there is considerable interest in the roles of the UDP-glucuronosyltransferase (UGT) family, which has led to widespread investigations of their potential effects in health and disease (1–4). In particular, genetic and chemical modification of UGT activity relating to steroid metabolism has ramifications for a range of pathologies such as prostate cancer, medication contra-indications, disruption to the endocrine system, and potential confounding effects on doping tests in sport. Therefore, it is timely to review lifestyle factors that affect UGT activity. Variations in the activity of UGT isozymes occur as a result of gender and ethnic origins giving different levels of expression of UGT forms and altered ratios of testosterone/epitestosterone excreted in urine (5). In addition to genetic variations, from a steroid metabolism viewpoint, one current focus of investigation is on the regulation of specific UGT activity via induction or inhibition by exogenous compounds such as pharmaceuticals and dietary components.

Several reports show induction of UGT activity by a range of compounds including phytochemicals and pharmaceuticals (6–8). Early studies reported the effects of drugs and dietary compounds on UGT activity in isolated microsomes or in rats without detailing the specific UGT isozymes involved (9, 10). Liver microsomal glucuronidation of estradiol and estrone was inhibited by green and black teas, along with a constituent catechin [(-)-epigallocatechin gallate] and several flavonoids (kaempferol, quercetin, rutin, flavone, naringenin, hesperetin) (11). Green tea polyphenols had a strong inhibitory effect of glucuronidation *in vitro* and showed a small increase in liver glucuronidation activity against estrone and estradiol was observed *in vitro* in rats with green tea as the sole fluid source (11). Consequent alterations in steroid metabolism have been debated to have a range of putative effects including varying responses to doping tests, inter-medication interactions, and susceptibility to developing cancer (2, 12).

From a treatment perspective, the roles of common compounds, including dietary components have been investigated as UGT inhibitors with a view to enhancing bioavailability of drugs. This approach to impairing metabolism and thus increasing the half-lives of drugs has been the subject of patent protection for a wide range of drugs (raloxifene, 2-methoxyestradiol, irinotecan, estradiol, labetalol, dilevalol, zidovudine, and morphine) using numerous inhibitors from plant origin (epicatechin gallate, epigallocatechin gallate, octyl gallate, propyl gallate, quercetin, tannic acid, benzoin gum, capsaicin, dihydrocapsaicin, eugenol, gallocatechin gallate, geraniol, menthol, menthyl acetate, naringenin, allspice berry oil, *N*-vanillylnonanamide, clovebud oil, peppermint oil, silibinin, and silymarin) (13). Regulation of UGTs by phytochemicals has been reviewed with a focus on cancer prevention (3).

The aim of this review is to present a critical evaluation of the current literature on dietary effects on steroid clearance. To date, the reports have focused on *in vitro* studies using supersomes, microsomes, and enzymes model systems, with reports of *in vivo* studies with a focus on UGT2B17 are lacking. Thus, it is apposite to generate a fuller understanding of the role of dietary components before *in vivo* studies are undertaken.

## Pharmaceutical Inhibitors of UGT Steroid Glucuronidation

Early reports demonstrated that a number of compounds interfere with the activity of UGT2B17 which is the major isozyme for clearance of anabolic steroids, having greater than double the activity of the next most active form UGT2A1. Sten et al. (14, 15) reported that epitestosterone and two non-steroidal anti-inflammatory drugs (NSAID) act as competitive inhibitors against UGT2B17. Using human microsomes and recombinant enzymes they demonstrated that diclofenac and ibuprofen inhibited testosterone glucuronidation without having significant effects on epitestosterone glucuronidation. Similar inhibitory effects on testosterone glucuronidation were reported for both UGT2B15 and UGT2B17 isozymes in *in vitro* studies. The authors measured IC_50_ values for diclofenac inhibition of testosterone glucuronidation by UGT2B15 and UGT2B17 of 25 μM and 65 μM respectively, at testosterone concentrations of 10 μM. The corresponding IC_50_ values for ibuprofen were 121 μM and 1340 μM against UGT2B15 and UGT2B17 respectively. Kinetic experiments using Dixon plots revealed that the diclofenac acts through competitive inhibition.

**Figure 1:**
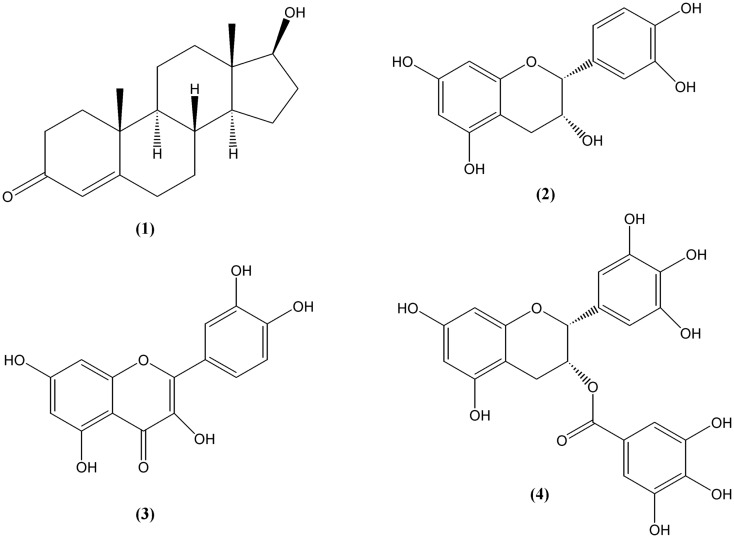
**Structures of testosterone (1) and selected inhibitors: epicatechin (2), quercetin (3), and epigallocatechin gallate (4)**.

To date, no commensurate studies have been reported demonstrating an effect of pharmaceuticals on testosterone glucuronidation *in vivo*. A recent report showed only a slight modification but no significant effects of concomitant use of maximum recommended doses of ibuprofen or diclofenac with testosterone on the urinary ratios of testosterone/epitestosterone in individuals with either two, one, or no allele of the UGT2B17, and no effect when ibuprofen/diclofenac was administered prior to single dose of testosterone (16). Given the competitive nature of the inhibition, at least for diclofenac, the experiment was limited by restriction to maximum doses of the NSAID. Thus, doses of 50 mg × 3 per day of the single competitive inhibitor, although well reasoned, may not elicit an inhibitory effect given that ibuprofen can also elevate UGT enzyme activity *in vivo* (8). Although reports of *in vivo* studies are lacking to date, the potential effects of inhibiting major testosterone-metabolizing enzymes warrants further exploration, especially if common substances are considered where maximum dosage effects do not limit intake. From one standpoint, this effect could alter the results of a doping test which is based on the ratio of the glucuronidated testosterone and epitestosterone. Following these advances, researchers have recently explored the effects of dietary components on steroid metabolism. The chemical structures of testosterone and selected inhibitors are shown in Figure 1.

## Dietary Inhibitors of UGT Steroid Glucuronidation

Given the growing body of literature regarding: (i) key roles for UGT enzymes in the metabolism a wide range of endogenous and exogenous compounds, (ii) the increasing understanding of the specificity UGT isozymes for varying substrates, and (iii) the roles of many common substances in elevating UGT activity *in vivo* and reducing UGT activity *in vitro*; studies on the roles of dietary components on testosterone glucuronidation *in vitro* were warranted.

Jenkinson et al. (17) first reported the effects of dietary green and white teas on the activity of UGT2B17 toward testosterone glucuronidation. Using an high performance liquid chromatography (HPLC) assay, testosterone glucuronidation was monitored in the presence of tea extracts using human UGT2B17 supersomes. Under the conditions studied, green and white tea preparations inhibited the reaction by circa 20% with a white tea powder inhibiting glucuronidation by 30%. HPLC analysis of the teas revealed key constituents such as epicatechin (EC) and epigallocatechin gallate (EGCG). Analysis via a Dixon plot revealed that EGCG was acting as a competitive inhibitor with an IC_50_ value of 64 μM which equaled that found previously for diclofenac (15). At a concentration of 1 mM, EC inhibited testosterone (at 10 μM) glucuronidation by some 55% (17).

Further studies by the authors, using a different HPLC method, revealed that cacao also inhibits UGT2B17 but to a lesser extent (ca. 15%) as shown in Table 1 (18). Under these conditions, at testosterone concentrations of 12 μg/mL, white and green tea preparations inhibited over 70% of activity with a white tea powder form showing inhibition of some 90% (Table 1). For the individual phenolics, inhibition was insignificant for gallocatechin and caffeine but ranged up to 22 and 42% for (−) epicatechin and (+) epicatechin respectively (Table 1). As shown in Table 1, extensive inhibition of testosterone glucuronidation was observed for epicatechin gallate (ca. 70%), epigallocatechin gallate (ca. 78%), and catechin gallate (ca. 90%) (17).

**Table 1:** **Inhibitory profiles for intact foods and catechins**.

Foods	Testosterone glucuronidation rate (ng/mL/min/mg protein)
Testosterone control (12 μg/mL)	682.09 ± 30.73
Cacao beans	666.22 ± 23.55
Cacao block	572.89 ± 20.14
White tea beard	249.83 ± 18.87
White tea leaf	246.22 ± 16.61
Green tea	179.56 ± 22.64
White tea powder	69.57 ± 11.04

**Catechin (250 μM)**	**Testosterone glucuronidation rate (ng/mL/min/mg protein)**

Testosterone control (10 μg/mL)	453.77 ± 10.24
Gallocatechin	446.25 ± 37.92
Caffeine	441.25 ± 23.75
Epigallo catechin	420.42 ± 27.08
(−) Epicatechin	352.08 ± 20.42
(+) Epicatechin	264.17 ± 15.83
Epicatechin gallate	143.75 ± 13.75
Epigallocatechin gallate	99.17 ± 19.17
Catechin gallate	70.42 ± 7.92

Analysis of the tea and cacao samples by HPLC (17, 18) revealed the catechins displayed in Table 1 were present in these samples at lower levels in comparison to the tea samples. The cacao samples, whilst inhibiting testosterone glucuronidation, did so at a much lesser rate than the tea samples which could be linked with having lower levels of inhibiting catechin compounds at the same concentrations of the tea samples.

In addition, red wine and its constituents were shown to inhibit testosterone glucuronidation by human UGT2B17 supersomes (19). Under the conditions studied, red wine inhibited glucuronidation by up to 70% over a 2-h period, with little effect arising from the alcohol content. Phenolic components were selected following HPLC analysis of the selected red wine and quercetin, caffeic acid, and gallic acid inhibited UGT2B17 testosterone glucuronidation by 72, 22, and 9% respectively, with concentrations of phenolic:testosterone of 100 μM:250 μM. For the most active phenolic, reducing the quercetin concentration to 2 μM, maintained inhibition of 20% in spite of the 10-fold excess of testosterone.

## Discussion and Future Directions

Based on the observed effect *in vitro*, the presence of flavonoids and catechins in a wide range of foodstuffs points to the potential for interaction *in vivo* with UGT2B17 activity. However, to our knowledge this has yet to be investigated in a clinical setting. Studies have shown oral administration of green tea extract catechins in rats lead to increased plasma testosterone as well as elevating other hormones over 8 weeks (20). The short term effects of iP administration EGCG in rats over 7 days found a number of hormones in serum dropped including testosterone, however hormone levels remained the same when EC, epigallocatechin (EGC), and epicatechin-3-gallate (ECG) were administered (21).

Dietary components inhibiting UGT2B17 could have clinical significance in altering the risk for prostate cancer. A recent review (2) highlights a number of studies that demonstrate an increase risk of prostate cancer with altered UGT2B17 function, although in the midst of conflicting evidence, determining the consequence of UGT2B17 polymorphism in prostate cancer risk has remained inconclusive. In a clinical setting catechins have been analyzed more for their apoptotic activities on prostate cancer cells rather than the links between endocrine levels and prostate cancer (22).

Clearly, the confounding issue of the contrasting effects of elevating enzyme activity, perhaps by induction, and concomitant chemical inhibition of the enzyme, will require rigorous investigation. We need to considerably further our knowledge of the effects of diet on the key UGT isozymes involved in steroid metabolism. This includes effects and mechanisms leading to elevation of enzyme activity but also gaining a full profile of inhibitors. Given the potential for varied responses in different tissues, the roles of UGT2B17 inhibitors as endocrine disrupters (23), developing androgen related pathologies, and in contra-indications to medicines still warrants full investigation.

## Conflict of Interest Statement

The authors declare that the research was conducted in the absence of any commercial or financial relationships that could be construed as a potential conflict of interest.
